# Chia Seeds (*Salvia Hispanica* L.): An Overview—Phytochemical Profile, Isolation Methods, and Application

**DOI:** 10.3390/molecules25010011

**Published:** 2019-12-18

**Authors:** Maša Knez Hrnčič, Maja Ivanovski, Darija Cör, Željko Knez

**Affiliations:** 1 Faculty of Chemistry and Chemical Engineering, University of Maribor, SI-2000 Maribor, Slovenia; maja.ivanovski@gmail.com (M.I.); darija.cor@um.si (D.C.);; 2 Faculty of Medicine, University of Maribor, SI-2000 Maribor, Slovenia

**Keywords:** chia seed, nutritional properties, active compounds, antioxidant activity, extraction methods

## Abstract

Chia (*Salvia hispanica* L.) is a small seed that comes from an annual herbaceous plant, *Salvia hispanica* L. In recent years, usage of Chia seeds has tremendously grown due to their high nutritional and medicinal values. Chia was cultivated by Mesopotamian cultures, but then disappeared for centuries until the middle of the 20th century, when it was rediscovered. Chia seeds contain healthy ω-3 fatty acids, polyunsaturated fatty acids, dietary fiber, proteins, vitamins, and some minerals. Besides this, the seeds are an excellent source of polyphenols and antioxidants, such as caffeic acid, rosmarinic acid, myricetin, quercetin, and others. Today, chia has been analyzed in different areas of research. Researches around the world have been investigating the benefits of chia seeds in the medicinal, pharmaceutical, and food industry. Chia oil is today one of the most valuable oils on the market. Different extraction methods have been used to produce the oil. In the present study, an extensive overview of the chemical composition, nutritional properties, and antioxidant and antimicrobial activities, along with extraction methods used to produce chia oil, will be discussed.

## 1. Introduction

*Salvia hispanica* L., also known as chia, is an annual herbaceous plant, originally from Southern Mexico and Northern Guatemala. It belongs to the order Lamiales, mint family Labiate, subfamily Nepetoideae, and genus *Salvia*. The genus *Salvia* consists of approximately 900 species, which have been widely distributed for thousands of years around several regions of the world, including Southern Africa, Central America, North and South America, and South-East Asia [1,2,3,4,5,6,7,8]. As reported in the literature, chia today is not only cultivated in Mexico and Guatemala, but also in Australia, Bolivia, Columbia, Peru, Argentina, America, and Europe. Nowadays, Mexico is recognized as the world’s largest chia producer [2].

Historical records testify that *Salvia hispanica* L. was used beside corn, bean, and amaranth by ancient Mesoamerican cultures—Aztecs and Mayas—in the preparation of folk medicines and food. In pre-Columbian societies, it was the second main crop after beans [3]. In the Aztecs communities, chia was used for food, cosmetics, and religious rituals.

*Salvia hispanica* L. is mainly grown for its seeds and produces white and purple flowers, which are 3 to 4 mm small and hermaphrodites. The plant itself is sensitive to daylight, it can grow up to 1 m tall, its leaves are reverse petiolate and serrated, and are 4 to 8 cm long and 3 to 5 cm wide. Chia seeds are generally very small, oval-shaped, 2 mm long, 1 to 1.5 mm wide, and less than 1 mm thick [2,5,6,9]. The color of the seed varies from black, grey, or black spotted to white. As Knez Hrnčič et al. [9] already reported, there is such a marginal difference between black and white Chia seeds that most consider them equal. Nutritional values are similar—protein content in black Chia seeds is 16.9% and fiber content is 32.6%. In white Chia seeds, the protein content is reported to be 16.5% and the fiber content 32.4%. A slight difference is only in morphology—white seeds are larger, thicker, and broader compared to black seeds. It is worth to mention that when black chia seeds are cultivated, around 5% to 8% of white chia seeds are grown at the same time. Cultivating only white chia seeds gives white chia seeds only.

Moreover, the plant itself can produce 500 to 600 kg seed/acre under appropriate agronomic conditions [3].

In recent years, Chia seeds have become one of the world’s most recognizable foods based on their nutritional properties and medicinal values [3,5,6,7,10]. Coorey et al. [11] reported that Chia is an excellent ingredient since it contains the highest known amount of α-linolenic acid and can be easily added to commercial food. It has been reported in several studies that chia seeds—due to the high percentage of fatty acids present—can be crucial for health, antioxidant, and antimicrobial activity [3,6,12,13,14].

Furthermore, the word chia comes from the Spanish word “chian”, which means oily. According to different sources [1,10,15], chia is an oilseed, with a powerhouse composed of fats, carbohydrates, dietary fiber, proteins, vitamins (A, B, K, E, D), minerals, and antioxidants. The advantages of using chia seeds as a nutritional supplement are enormous—positive benefits include supporting the digestive system, promoting healthy skin, stronger bones and muscles, reducing the risk of heart disease, diabetes, and so on [2,3,5,16]. It contains a high number of polyphenolic antioxidants; the seed is free from mytoxins and it does not contain gluten [3].

Lately, there have been many new discoveries regarding the nutritional properties, phytochemicals, and extraction methods regarding chia seeds. The aim of this study is to present these findings in the nutritional and therapeutic potential of chia seeds, focusing on the extraction methods used.

## 2. Chemical Composition and Phytochemicals in Chia Seeds

**Figure 1:**
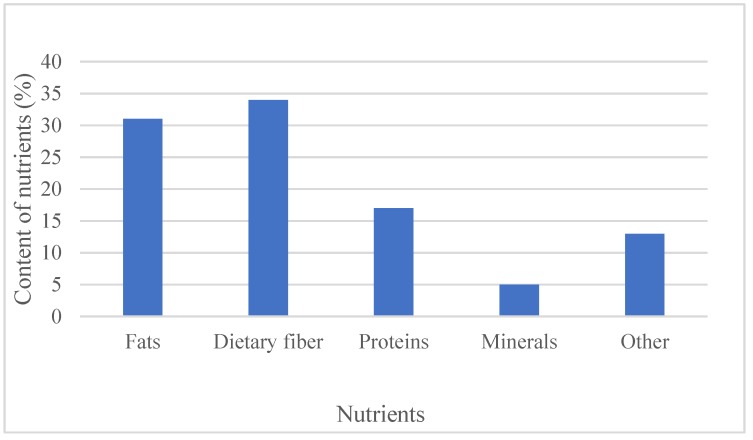
Basic composition of chia seeds [19].

The chemical composition of chia seeds have been analyzed by many researchers [3,5,6,17]. Chia seeds contain a high content of fats (30–33%), carbohydrates (26–41%), dietary fiber (18–30%), proteins (15–25%), vitamins, minerals, and antioxidants (wet basis) (Figure 1). Table 1 shows the nutritional profile of chia seeds in 100 g as stated by the National Nutrient Database of the USDA [18], and the comparison of its properties with other well-known cereals. Many researches on the phytochemicals have been reported, highlighting that the major constituents of chia oil are polyunsaturated fatty acids (PUFAs: α-linolenic (ALA, ω-3 fatty acid) and linoleic (LA, ω-6 fatty acid) acids) [10]. Chia seeds contain 39% oil (mass of dry seed), which consists up to 68% of ω-3 and 19% of ω-6 fatty acid [1,5]. The ratio between ω-6 and ω-3 fatty acid is 0.3:0.35 [19]. Campos et al. [4] and Coates and Ayerza [17] stated that the chemical composition of each product can vary due to different factors such as year of cultivation, environment of cultivation, and extraction method used. Coates and Ayerza [20] as well investigated the effect of temperature on the polyunsaturated fatty acids present in chia seeds in Argentina. Their results have shown that during seed development, from April to May, the increase of the temperature decreases the amount of polyunsaturated fatty acids (PUFAs) present. According to the source in the literature, PUFAs are essential for human health, but cannot be synthesized by the human body itself, only with diet does the human body receive them [21]. Furthermore, Musa Özcan et al. [22] investigated how microwave heating treatments at different powers are effecting the physicochemical properties of chia seeds, including phenolic content, antioxidant activity, and fatty acid composition. Results have shown that roasting chia seeds in the microwave causes changes in the chemical composition of the chia oil (such as varying the content of α-linoleic acid or caffeic acid when using different powers).

The benefits of ω-3 fatty acid on the human body include the following: lowering the content of three glycerides and cholesterol levels, anti-inflammatory activity, cardioprotective and hepatoprotective activities, antidiabetic action, and protection against cancer, arthritis, and autoimmune disease. Meanwhile, the benefits of ω-6 include anti-inflammatory activity, anti-hypertensive, anti-thrombotic activities, and anticancer activities [2,3,5,6,16].

### 2.1. Protein Content

The protein content of chia seeds is around 17%, greater than the protein content in all other cereals (for instance, in corn the protein content is 9.4%, rice is 6.5%, quinoa 14.1%, and in wheat 12.6%) [3,23,31]. The amount of proteins in chia seeds depends mainly upon environmental and agronomical factors [3]. The U.S. Department of Agriculture [19] has confirmed that chia seeds contain some exogenous amino acids (arginine, leucine, phenylalanine, valine, and lysine) and some endogenous amino acids (glutamic and aspartic acid, alanine, serine, and glycine). For example, the content of amino acid serine is 1.05 g/100 g, glutamic acid 3.50 g/100 g, glycine 0.95 g/100 g, alanine 1.05 g/100 g, lysine 0.97 g/100 g, and histidine 0.53 g/100 g [3]. The absence of the protein gluten makes chia seeds highly valued to patients suffering from celiac disease. Moreover, food rich in proteins is highly recommend to people who are fighting to lose weight. Grancieri et al. [2] was investigating the composition and positive effects of chia seeds, proteins, and peptides, and their effect on the human body. A total of 20 proteins were obtained from chia seeds, eight of them were specially related to the production of the plant lipids, which cause the high concentrations of polyunsaturated fatty acids presented. The authors suggested further in vitro and in vivo investigations to investigate the properties of chia proteins. Coelho and Salas-Mellado were investigating how the choice of extraction methods effects the physical and functional properties of chia proteins [32]. The following methods were used: two methods of obtaining protein concentrates (chemical methods, CPC1, CPC2) and one method for obtaining a rich fraction (dry fractionation method, PRF). The protein content from the extracts were 70.9%, 74.1%, and 49.7%, respectively. The results have shown that the optimal method used for investigating the protein content in chia seeds depends on the desired product. Following this, Urbizo-Reyes et al. [33] investigated how the biological and functional properties of chia seeds’ protein hydrolysate improves when using ultrasonication to remove mucilage and how microwave-assisted enzymatic hydrolysis are generating bioactive and functional chia seeds’ peptide.

### 2.2. Fibre Content

The fiber content in chia seeds is also very high. Chia seeds contain between 34 g and 40 g of dietary fiber per 100 g [3,6]. In this specific amount, the insoluble fraction (IDF) represents approximately 85–93%, while the soluble fraction (SDF) represents between 7% and 15% [19] (Table 4). Reyes-Caudillo et al. [13] showed in their research that a high amount of fiber decreases the risk of coronary heart disease, risk for diabetes type 2, and several types of cancer, and a high amount of dietary fiber in daily meals decreases subsequent hunger.

### 2.3. Minerals

Additionally, chia seeds contain minerals like calcium, phosphorus, potassium, magnesium, and vitamins (A, B, K, E, D, mainly vitamins B1, B2, niacin) (Table 1). The content of calcium, for instance, is greater than in rice, barley, corn, and oats.

The content of other minerals such as magnesium, potassium, and phosphorus is greater in chia seeds as well than in other cereals [3,31].

### 2.4. Phenolic Compounds

Focusing on phenolic content, dry chia seeds contain 8.8% of phenolic compounds. Besides that, high levels of caffeic acid, chlorogenic acid, querencetin, rosmarinic acid, gallic, cinnamic, myricetin, kaemferol are also reported. Furthermore, isoflavones, such as daidzein, glycitein, and genistein, are found in small amounts (Table 2). Rahman et al. [34] reported that rosmarinic acid and daidzein are the major components found in chia seeds, along with caffeic acid, mycertin, quercetin, etc. Besides, in the same study, vitamins A, B1, B2, and B3 were identified in chia seeds for the first time. The flavonoids quercetin, chlorogenic acid, and caffeic acid are proven to have anti-cancerogenic, anti-hypertensive, and neuron protective effects [5]. Both Mohd Ali et al. [6] and Ullah et al. [3] mentioned that chia seeds have no potentially toxic mycotoxins or gluten present. Furthermore, Uribe et al. [35] described that chia seeds are a great example of a food rich in antioxidants. Reyes-Caudilio et al. [13] also stated that chia seeds are a great source of antioxidants with a wide range of antioxidant compounds. Martinez-Cruz and Paredes-Lopez [36] used chia seeds for analyzing total phenolic contents, antioxidant activity, and qualification of phenolic acids and isoflavones by using ultra high performance liquid chromatography (UHPLC). With this method, major phenolic compounds were measured—rosmarinic acid, caffeic acid, and daidzein. All the active compounds in *Slavia hispanica* are presented in Table 2.

Some researchers reported that the chemical composition and nutritional values may vary due to climatic conditions, geographic location, nutrients, and year of cultivation [6]. For example, the composition of fatty acids may vary according to climate change and the altitude of the plant—the colder and higher the region, the higher the content of ω-3 unsaturated fatty acid.

## 3. Antioxidant and Antimicrobial Activity

Chia seeds and their oil contain a large number of natural antioxidants, such as tocopherols, phytosterols, carotenoids, and polyphenolic compounds, which have already been discussed in Section 2. Polyphenolic compounds are the most important complexes that contribute to the antioxidant activity of chia seeds. It is well known that they have the ability to scavenge free radicals, to chelate ions, and to donate hydrogens [7]. Antioxidant compounds reduce the risk of chronic diseases (cancer and heart attack) and they offer protection against some disorders such as diabetes, Alzheimer’s, and Parkinson’s disease [2]. ω-3 fatty acids have the capability to block calcium and sodium channel dysfunctions, which can cause hypertension, as well as improve heart rate variability and protect ventricular arrhythmia [37].

Antioxidant activities were also investigated by Sargi et al. [38] and Clau-Solis et al. [1]. Among the analytical techniques, for the determination of antioxidant activity, ABTS^+^ (monocationic radical from ABTS diammonium salt), DPPH (2,2-dyphenyl-1-picrylhydrazyl), and FRAP (fluorescence recovery after photobleaching) have been applied most recently. Sargi et al. [38] investigated the antioxidant activity of chia seeds from Mexico and Argentina by using the above-mentioned techniques. Authors have considered that chia seeds are capable of deactivating ABTS^+^ cation radicals. The same authors also showed that chia seeds exhibit the capacity to scavenge synthetic DPPH radicals and reduce iron ions. The same results were obtained by other authors such as Clau-Solis et al. [1] and Reyes-Caudillo et al. [13]. Reyes-Caudillo et al. [13] investigated the antioxidant activity of phenolic compounds in chia seeds. Chia seeds from two different regions in Mexico were extracted. The ABTS^+^ radical scavenging method, together with β-carotene linoleic-acid principle and phospholipid liposome peroxidation, was used in research to determine antioxidant activity, whilst Guindani et al. [39] used the ABTS^+^ method to determine antioxidant activity as well. Alacantara et al. [40] investigated antioxidant activity by the DPPH method.

Grancieri et al. [2] stated in their research that to investigate the specific antioxidant activity, further in vitro studies should be carried out.

Several authors investigated the positive effects of the polyphenolic compounds in chia seeds using different analytical techniques. Chemical compounds, such as caffeic acid, ferulic acid, chlorogenic acid, rosmarinic acid, and flavonoids (quercetin, kaempferol, daidzein, etc.), have been mainly investigated by different analytical techniques, where UHPLC (ultra-high performance liquid chromatography), HPLC (high performance liquid chromatography), and UPLC (ultra-performance liquid chromatography) particularly stand out. Their biological activities vary from antioxidant, anti-aging, and anti-hypertensive to anti-cancerogenic and anti-inflammatory.

In comparing chia seeds to other cereals mentioned in this work, the antioxidant activity of rice, corn, wheat, quinoa, and amaranth have been also investigated and reported [41,42,43,44,45].

## 4. Extraction Methods

Over several years, several extraction methods, techniques, and solvents, were used for producing thechia oil. Intense research has been performed due to development of the concepts of green chemistry, which demands the usage of less harmful solvents for extraction [10]. However, detailed studies to characterize the oil and investigate the influence of different extraction methods and conditions on antioxidant activity are still limited. Silva et al. [10] stated that due to chia oil’s primary usage in the food industry, the extraction solvents must be compatible with the requirements of the food industry. It is known that the usage of different extraction methods are causing variation in the extraction yields, quality, and content of fatty acids, as well as the content of dietary fibers, antioxidant content, etc. Ciau-Solis et al. [1] and Knez Hrnčič et al. [9] report the extraction of chia oil conventionally by Soxhlet extraction. Non-polar conventional organic solvents such as *n*-hexane or ether are used. The advantages of using conventional solvent (CS) extraction are mainly the simplicity of the method, relatively high extraction yield, and suitable functional characteristics of the oil (such as water holding, absorption capacity, organic molecule absorption, molecule stability). Meanwhile, the disadvantages are decreased antioxidant activity, due to the decomposition of thermolabile antioxidants, and the environmental and health concerns involved in using *n*-hexane.

Silva et al. [10] were studying the properties of chia oil extracted by using different solvents (ethyl acetate, isopropanol, and *n*-hexane). It was considered that in relation to the oil extraction, higher yields were obtained with *n*-hexane and ethyl acetate. Moreover, the solvent properties did not affect the properties of the oil, and the water-holding capacity was not affected by the extraction process.

A more suitable extraction method that has been used recently is supercritical fluid extraction (SFE), where carbon dioxide (CO_2_) is the most used solvent [46]. Entering into the supercritical state depends on both temperature and pressure. A “supercritical” solvent exhibits the features of both a liquid and a gas. It can slide into porous materials as well as dissolve them. The advantages of using SFE comparing to other techniques are the usage of a solvent with low density, viscosity, surface tension, and mild conditions of temperature and pressure, which leads to no degradation of the compounds. Not only CO_2_, but also other solvents can be used for SFE. Meanwhile, CO_2_ has beneficial properties such as a low temperature (31 °C) and low critical pressure (7.29 MPa). Ixtaina et al. [47] studied the effect of temperature, pressure, and time on the SFE-CO_2_ extraction of oil from Mexico based chia seeds. Authors obtained chia oil with the following characteristics: yield 92.8%, at 45 MPa, 80 °C, and 300 min. The same authors performed the same experiment on the material from a different origin. Chia seeds from Argentina were used in this study, where the authors obtained oil recoveries ranging from 82% (25 MPa, 40 °C, 285 min) to 97% (45 MPa, 60 °C, 138 min) [48]. Guindani et al. [39] investigated the oil extraction from chia seeds using SC-CO_2_ and ethyl acetate as co-solvents. The highest extraction yield, 10.6%, with pure CO_2_ was obtained at 30 MPa and 50 °C.

Supercritical extractions using CO_2_ are common for the extraction of Chia oil. Subcritical extractions are also performed regularly, mostly by using propane in its subcritical state. The differing methods accomplish different goals. The pressure is maintained, while the temperature is taken below the supercritical threshold. The solvent still maintains some of the hydrophobic extraction properties, but the lower temperatures protects the fragile constituents from denaturing. Silva et al. [49] and Knez Hrnčič et al. [9] performed the subcritical extraction of oil with *n*-propane. The work of Knez Hrnčič et al. [9] demonstrates that the extraction yield and composition of extracts from chia seeds are slightly affected by the different process parameters studied (pressure, temperature). Additionally, it has been demonstrated that the composition of both black and white chia seed extract is similar. The use of different extraction conditions resulted in differing extraction yields, but did not significantly affect the composition of the extract. Higher operating pressures contributed to higher extraction yields when operating at higher temperature. It has been demonstrated that the composition of oils, obtained from both seed varieties is similar. Brunner’s equation was employed to model extraction kinetic curves and the accuracy of the model with experimental data has been proven. The agreement between experimental and model data ranged from 3.13% to 7.27%. GC (Gas Chromatography) analyses confirmed presence of palmitic, stearic, oleic, linoleic, and linoleic acids. Linolenic acid is represented in the highest amount which increased with elevating operating pressure. Propane has been demonstrated as a suitable processing media for the extraction of oils with a high content of the two essential fatty acids (α-linolenic acid and linoleic acid). It can be concluded that the high operating pressure contributes to the higher extraction yield and the higher content of linolenic acid, the major compound in the extracted oils. The results suggest that the subcritical extraction with food grade n-propane is a potential method for obtaining high-quality chia seed oil, providing a product free of toxic residues, and therefore, suitable for subsequent use in food, pharmaceutical, and cosmetic industries.

Villanueva-Bermejo et al. [21] used a liquid-pressured extraction method for chia oil extraction with *n*-hexane, ethyl acetate, and aqueous ethanol as solvents. The authors extracted the chia oil with two different batches of Mexican chia seeds—high oil content seeds (HOCS), and low oil content seeds (LOCS). Supercritical fluid extraction (SFE) and pressured liquid extraction (PLE) were applied. No differences were found between the fatty acid profile of the oils extracted from HOCS and LOCS by both extraction methods. Chia seed oil was nanoencapsulated, utilizing chia seed mucilage as wall material [50]. The aim of the study was to evaluate if mucilage can be used for wall material, to characterize the chia seed oil nanoparticles, and determine its stability. The effect of different parameters such as temperature, the solvent to seed ratio, and extraction time in the ultrasound-assisted extraction (UAE) from chia seeds provided high yields in the extraction, and the chemical analyses indicated polyunsaturated fatty acids are the major oil components (82% of the total oil composition) by using ethyl acetate as a solvent. Ethyl acetate is considered a food-grade solvent, accepted in the food industry [51]. Extraction methods with extraction yields are presented in Table 3.

## 5. Applications of Chia Seeds and Derived Products

Over recent years, functional foods have gained remarkable consideration world-wide due to the wave of healthy lifestyle changes. Contemporarily, chia seed is used as a healthy oil supplement for humans and animals.

### 5.1. Food Industry

Several studies have been performed on the usage of chia seeds in the food industry. In the food industry, chia seeds can be used in different shapes: whole, ground, in the form of flour, oil, and gel [5]. In 2000, the US Dietary guidelines suggested that chia can be used as primary food, but in a limited amount; consumption of no more than 48 g/daily is recommended. Chia seeds can be added or mixed into biscuits, pasta, cereals, snacks, and cakes as supplements. Due to their hydrophilic properties, chia seeds can be used as substitutes for eggs and fat. They can absorb 12 times their weight in water [5].

Chia gel may be used as substitutes for oil or eggs in baked products. It was shown that chia oil can replace 25% of the egg in cakes [17].

The nutritional value of butter can be increased by mixing it with chia oil in a proportion from 6.5% to 25%, when the concentration of ω-3 fatty acid in chia fortified butter increases from 4.17% to 16.74% [3].

Besides, recent studies showed that mucilage from chia seeds can be used as a functional coating with improved functional properties [3].

#### 5.1.1. Chia Mucilage

Chia mucilage could be employed in the food industry as a foam stabilizer, a suspending agent, emulsifier, adhesive, or binder as a result of its water holding capacity, and viscosity. Recent studies showed that mucilage from chia seeds can be used as a functional coating with improved functional properties [3]. Compared with other hydrocolloids such as gum arabic, modified starch and cellulose, chia mucilage has a low EAI—emulsifying activity index. The mucilage showed a significant ability to stabilize emulsions; however, the potency was affected by the emulsion’s composition. The fact that the mucilage has such an ability to stabilize emulsions may be due to its capacity to adsorb in the solid or liquid interface and stabilize emulsions without chemical or enzymatic variations. Mucilage obtained from chia seeds is a novel source of polysaccharides and could potentially generate interesting polymer blends for edible films and coating.

Edible films based on polysaccharides are potential substitutes for synthetic packaging. Mucilage has the ability to form edible films, but they are very slight and brittle. The addition of plasticizers may be carried out to advance the mechanical characteristics of edible films. Different plasticizers, such as polyols, have been applied to increase the flexibility and workability of such films. Amongst the plasticizers, glycerol is one of the most widely applied in film-making techniques. Thechia mucilage hydrocolloid is an interesting constituent that may be applied for the design of new film-forming solutions. The addition of glycerol to extracted hydrocolloid from the chia seed to make chia mucilage films was essential to provide homogenous and flexible films and is crucial to achieve the suitable physicochemical, barrier, and mechanical properties. The solubility of chia mucilage films can be fine-tuned by the glycerol content and improved with a higher proportion of glycerol [61]. Water solubility in the chia mucilage films plasticized with different concentrations of glycerol increased considerably.

#### 5.1.2. Chia Gum

Chia seeds are believed to be a starting material in the food industry for their dietary fiber. Gum can be extracted from the dietary fiber fraction by using water as an additive to control viscosity, stability, and texture. The chemical composition, molecular structure, and the derived properties such as thermal stability or gelling ability represent important factors which determine the appropriateness of a polysaccharide in food and pharmaceutical industries. The gum is also stable at high temperatures, way up to 224 °C. Ciau-Solis et al. [1] investigated the chemical and functional properties of chia seed gum. They showed that chia gum contains 26.2% fat, and when submitted to fat extraction, two fractions of the gum can be produced: gum with fat (FCG) and gum partially defatted (PDCG). They confirmed that PDCG has a higher content of protein, ash, and carbohydrate than FCG. Chia seed gum is a novel promising material. However, due to limited information on the structural composition, it has not yet been industrially applied. Few investigations have been carried out on its thermal stability and functionality. Understanding of these characteristics will significantly broaden the potential of industrial application [62].

### 5.2. Pharmaceutical Use

Bilayer emulsions have potential as delivery systems of ω-3 fatty acids from chia oil which represents a high potential in pharmaceutical applications and the food industry since the emulsions can be used directly or subjected to a drying process to obtain powders. Due to the relative ease of synthesis and economic feasibility, conventional oil-in-water (O/W) emulsions are usually the first choice considered to deliver bioactive lipids. Chia oil can be incorporated into oil-in-water (O/W) emulsions as ω-3 fatty acid delivery systems in food matrices. Sodium caseinate content and lactose addition strongly influence the stability and rheological properties of chia O/W emulsions. A moderate stability of chia O/W emulsions and Newtonian behavior is achieved by stabilization with a certain amount of emulsifier. Chia O/W emulsions have demonstrated low levels of primary and secondary oxidation products [63]. In the study of M. Julio and co-authors [64], chia bilayer O/W emulsions were obtained by applying the layer-by-layer deposition technique. It consisted of the electrostatic deposition of a positively charged chitosan on negatively charged oil droplets. These were stabilized using modified sunflower lecithins (deoiled or phosphatidylcholine-enriched) in the presence or absence of maltodextrin.

A recent study [65] reports that spray dried chia seed oil (CSO) microcapsules were prepared by using chia seed protein isolate (CPI), chia seed gum (CSG), and a CPI-CSG complex coacervate as shell materials. The CPI-CSG complex coacervate was found to be suitable for the delivery of CSO to the intestinal stage of digestion, since almost all the unencapsulated oil was hydrolysed, whereas only 60% of the oil encapsulated in CPI-CSG shell was hydrolysed during in vitro digestion. It is reported that the leaves of *Salvia hispanica* L. contain an essential oil that comprises β-caryophyllene, globulol, β-pinene, α-humoleno, and widdrol. Those compounds are believed to have strong repellent characteristics to a wide spectra spectrum of insects [3].

## 6. Therapeutic Value

Therapeutic values of chia seeds have been reported as well. Some of them are presented in Table 4. For example, cardio-protective effects have been analyzed by Munoz et al. [15]. Α-linolenic acid plays a significant role in the formation of some vital biochemical compounds such as leukotrienes and thromboxanes, which are connected to numerous physiological functions in the human body [3]. Moreover, ω-3 fatty acid has the capability of blocking calcium and sodium channels disfunctions (which can cause hypertension), improving the parasympathetic tone, and protecting ventricular arrhythmia [3]. Furthermore, eating Chia seeds in during pregnancy helps to develop the retina and brain of the fetus.

Consequently, incorporating dietary fiber and a-linolenic fatty acids into the diet makes Salba-chia a prime contender in regulating body weight and possibly other comorbidities associated with diabetes. A study of Vuksan and co-workers demonstrated that supplementing 37 g/day of Salba-chia to an isocaloric diet improved major and emerging risk factors in type 2 diabetes, suggesting its cardioprotective potential while maintaining weight. A subsequent study by the same group demonstrated that Salba-chia acutely reduced postprandial glycemia when added to a meal, and prolonged satiety. Further investigations demonstrated that a 6 month addition of Salba-chia to a calorie-restricted diet, in conjunction with the standard medical care, resulted in small, but significant, weight loss in overweight and obese participants with type 2 diabetes [66]. A comparison of the effect of two seeds (flax (*Linum usitatissimum*) and Salba-chia (*Salvia hispanica* L.)) on postprandial glycemia and satiety scores showed that despite the similarities in nutritional composition, Salba-chia appears to have the ability to convert glucose into a slow-release carbohydrate and affect satiety to a greater extent than flax, possibly due to the higher fiber viscosity. Fifteen healthy participants (M/F: 5/10; age: 23.9 ± 3 years; BMI: 22.2 ± 0.8 kg/m^2^) were randomized to receive a 50 g glucose challenge, alone or supplemented with either 25 g ground Salba-chia or 31.5 g flax, on three separate occasions. Blood glucose samples and satiety ratings were collected at fasting and over 2 h postprandially. In addition, in vitro viscosity of the beverages was assessed utilizing standard rheological methodology. Both seeds appeared to differentially alter carbohydrate metabolism and satiety, with Salba-chia having a stronger effect than flax. The 39% reduction in blood glucose iAUC (incremental area under the curve) observed for ground Salba-chia in the current study is in line with the reductions from previous studies of 35% and 42% vs. control at a comparable dose of 24 g. In contrast, ground flax has not been previously shown to affect postprandial glycemia. Though there is a slight suspicion that high fiber seeds should be promoted for their nutritional properties, the current findings suggest that the criteria for selection should also include their rheological properties rather than their absolute fiber content. Namely, viscosity is considered as a measure of the fiber’s contribution to viscosity development, independent of fiber concentration [67]. The consumption of chia flour is consistently able to decrease the blood pressure in hypertensive individuals, even in patients previously treated with medications in a manner similar to the patients not using medications [68]. Despite the reduction in lipid peroxidation as effect of chia, there was no verification whether this effect would be accompanied by increased antioxidant capacity. The effectiveness of milled and whole chia seed in altering disease risk factors in overweight, postmenopausal women was studied using a metabolomics approach. 62 overweight (body mass index 25 kg/m^2^ and higher), nondiseased, nonsmoking, postmenopausal women, aged 49–75 years were included. The study was performed by means of analysis based on the 56 subjects who completed all phases of the study. As a prestudy, diet records and questionnaire responses to assess potential adverse effects and adherence to the supplementation regimen were administered, and again after 5 and 10 week supplementation.

The results of research performed over male Wistar rats disclosed that feeding chia seeds had a great declining effect on triglycerides and enhanced beneficial HDL cholesterol [69]. Additionally, feeding chia seeds resulted in a reduction of omega-6 in plasma, which consequentially resulted in a lower ω-6:ω-3 ratio and has a subsequent cardio-protective effect. The effect of feeding chia seed (50 g/day) to 12 healthy individuals for 30 days was investigated by Vertommen and co-workers. The diastolic blood pressure decreased from 66.1 to 61.5 mmHg with a significant decline in serum triglycerides, and no side effect was reported [18].

Other studies were carried out to investigate the therapeutic effects which demonstrate chia seeds as a potential source of several bio-active peptides, essential for the repair of damaged tissue and general well-being [70], as well as the control of dyslipidaemia [13]. Furthermore, investigations in chia seeds as an anti-inflammatory agent [71], antiplatelet, anti-carcinogenic, laxative, hypotensive, cardiac tonic, cardiovascular protector, treatment of anaemia, treatment of dermatitis, analgesic [72], antidepressant, antianxiety, vision and immune improver [56], and EPA and DHA improver in blood [73] were carried out. The appearance of celiac disease, constipation, and vasodilatation [74], as well as the risk of kidney disorders, may be decreased by complementary consumption of whole and ground chia along with chia oil.

## 7. Conclusions

Chia, *Salvia hispanica* L., is a plant species used since ancient times for dietary and medical purposes. Its products are small dry white and dark seeds.

Recently, there have been many discussions and studies about the health benefits and use of this seed. Chia seeds contain a high fat content, carbohydrates, dietary fiber, proteins, vitamins (A, B1, B2, and B3), minerals, and antioxidants. Furthermore, chia seeds contain the flavonoids quercetin, chlorogenic acid, and caffeic acid, which are proven to have anti-cancerogenic, anti-hypertensive, and neuron protective effects. Furthermore, chia seeds are a rich source of nutrients such as polyunsaturated omega-3 fatty acids that protect from inflammation, improve cognitive performance, and lower the level of cholesterol. Chia seeds contain antioxidant compounds that reduce the risk of chronic diseases (cancer and heart attack) and offer protection against some disorders such as diabetes, Alzheimer’s, and Parkinson’s disease. Moreover, the high amount of fiber decreases the risk of coronary heart disease, the risk for diabetes type 2, and several types of cancer.

Chia seeds are already used in the food and pharmaceutical industry. In the food industry, chia seeds can be used in different forms: as the whole seed, ground, in the form of flour, oil, and gel. Chia oil is one of the most valuable oils on the market today. Nanoemulsion-based delivery systems are prospective applications to encapsulate lipophilic bioactive components in food, personal care, cosmetics, and pharmaceutical applications. Chia seed oil nanoemulsion delivery systems represent a possibility for the further application of chia seed oil in beverages and the functional food industry which requires only a slightly turbid or even transparent appearance. Chia seed mucilage represents a promising alternative to synthetic polymers in nanoencapsulation.

Different extraction methods and solvents such as Soxhlet extraction using *n*-hexane, ethyl acetate, and aqueous ethanol, supercritical fluid extraction with CO_2_ and pressured-liquid extraction have been proposed to obtain the oil. The compression method comprises pressing the seeds at 4 °C or 25 °C in dark. This contributes to the preservation of antioxidants; nevertheless, oil recovery is low. Soxhlet extraction using *n*-hexane is advantageous due to functional characteristics like absorption capacity and emulsifying stability. On the other hand, this method is least preferred as it poses health issues from the use of hexane. Sub and supercritical fluid extraction are currently the most promising alternatives that yield a better purity of ALA. The oil yield increases with pressure elevation. The supercritical fluid extraction (SFE) and the pressured liquid extraction (PLE) of oil was performed from high oil content seeds (HOCS) and low oil content seeds (LOCS). The results show no difference in the fatty acid profile of the oils extracted from HOCS and LOCS by both extraction methods.

Despite several epidemiological and experimental reports promoting the medicinal use (oral supplements) of chia, protocols regarding the extraction and effective dose should be standardized in order to suit human consumption on a large scale, supported by sound scientific data. Dietary chia provides an array of pharmacological properties, but knowledge and understanding of the bioactive and fatty acids responsible for its biological activity using mechanistic approaches in cell and mammal models are still the main limitations for its wider therapeutic usage.

## Figures and Tables

**Table 1:** Nutritional properties, vitamins, fatty acids, and phenolic compounds content of chia seeds and other cereals per 100 g [3,5,23,24,25,26,27,28,29,30,31].

	Chia Seeds	Rice	Corn	Wheat	Quinoa	Amaranth
**Carbohydrates (g)**	42	80	74	71	64.2	71
**Protein (g)**	17	6.5	9.4	12.6	14.1	12.6
**Fat (g)**	31			1.5	1.92	1.5
**Minerals (mg)**						
Magnesium	335	25	127	126	197	126
Phosphorus	860	115	210	288	457	288
Calcium	631	28	7	29		29
Potassium	407	115	287	363	563	363
Natrium	16	/	/	/	/	/
**Other (g)**	13	/	/	/	/	/
**Vitamins (mg)**						
Vitamin A eq.	54 μg	0	214	9	0	n.d.
Vitamin E	0.5	0.11	0.49	1.01	0.63	1.19
Vitamin C	1.6	0	0	0	0	4.2
Thiamine (B1)	0.62	0.07	0.39	0.30	0.11	0.12
Riboflavin (B2)	0.17	0.05	0.20	0.12	0.11	0.2
Niacin (B3)	8.83	1.6	3.63	5.46	0.412	0.92
**Fatty acid content (%)**						
Linolenic acid (C18:3, ω-3)	63.79	2.1	1	0.08	6.7	1.01
Linoleic acid (C18:2, ω-6)	18.89	39.7	52	0.68	56.4	0.35
Olec acid (C18:1, ω-9)	7.3	35.1	31	0.24	20.4	22.69
Palmitoleic acid (C16:1)	0.03	/	/	/	n.d.	0.08
Eicosenic acid (20:1)	n.d.	/	/	0.005	n.d.	1.49
Palmitic acid (C16:0)	7.04	20.8	13	3.02	9.7	18.59
**Phenolic compunds (μg)**						
Caffeic acid	27	n.d.	26	40	37	0.90
Quercetin	0.17	/	/	30.1	43.3	/
Kaempferol	0.013	/	/	/	36.7	/
Daidzin	6.6	/	/	/	/	/
Glycitin	1.4	/	/	/	/	/
Genistin	3.4	/	/	/	/	/

**Table 2:** Active compounds in *Salvia hispanica* L.

Active Compounds in *Salvia Hispanica* L. Seeds	Chemical Structure	Biological Activity	Reference
Omega-3 fatty acid, ω-3 fatty acid, ω-3 ALA	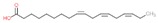	- anti-inflammatory	[6]
		- antidiabetic	
		-anticancer	
Omega-6 fatty acid, ω-6 fatty acid, ω-6 LA	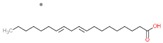	- inflammatory	[6]
		- anticancer	
*Flavonoids*			
Mycertin	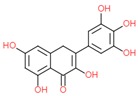	- antioxidant	[6]
			[7]
			[34]
Quercetin	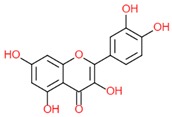	- antioxidant	[7]
		- anti-cancerogenic	[6]
		- anti-hypertensive	[34]
Kaempferol	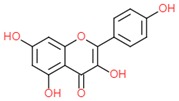	- antioxidant	[6,7]
			[6]
Caffeic acid	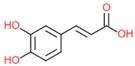	- antioxidant	[7]
		- anti-cancerogenic	[34]
		- anti-hypertensive	[36]
Rosmarinic acid	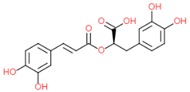	- antioxidant	[7]
			[34]
			[36]
Chlorogenic acid	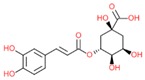	- antioxidant	[34]
		- anti-cancerogenic	
		- anti-hypertensive	
Vitamins	A	- healthy skin	[34]
	B1	- for synthesizing ATP	[34]
	B2	- for normal red blood cells working	[34]
	B3	- for normal nervous and digestion system working	[34]

**Table 3:** Extraction methods, extraction yields, and fatty acid content.

	Methods of Extraction	Solvent	Extraction Yield (%) ^a^		Fatty Acid Content (%)^a^				Reference
			White Chia Seeds	Black Chia Seeds	ω-3		ω-6		
					White Chia Seeds	Black Chia Seeds	White Chia Seeds	Black Chia Seeds	
1	Cold solvent extraction	*n*-hexane	30.0	30.0	Not evaluated	3.5	4.0	2.97	[23]
2	Cold solvent extraction	*n*-hexane	42		Not evaluated		Not evaluated		[52]
3	Cold solvent extraction	*n*-hexane	19.3		67.9		17.6		[53]
4	Soxhlet extraction	*n*-hexane	13.8		62.8		20.1		[39]
		ethyl acetate	12.10		Not evaluated		Not evaluated		
		ethanol	15.4		64.1		19.9		
5	Soxhlet extraction	*n*-hexane	Not evaluated	Not evaluated	48.52	48.66	17.98	17.98	[9]
6	Soxhlet extraction	*n*-hexane	10.9		Not evaluated		Not evaluated		[1]
7	Soxhlet extraction	*n*-hexane	34.6	35.6	Not evaluated	3.5	4.0	2.97	[23]
8	Soxhlet extraction	*n*-hexane	25.7–32.2		54.4–54.4		20.2–21.8		[46]
9	Soxhlet extraction	*n*-hexane	Not evaluated		Not evaluated		Not evaluated		[12]
10	Soxhlet extraction	*n*-hexane	26.7–33.6		65.6–69.3		16.6–19.7		[48]
11	Soxhlet, Ultrasonic extraction	*n*-hexane, ethyl acetate, isopropanol	33.6		62.4		19.6		[10]
			30.2		62.4		19.4		
			25.6		62.9		19.8		
12	Ultrasonic extraction	*n*-hexane	Not evaluated		51.5	46.4	19.5	19.5	[9]
13	Ultrasound extraction	*n*-hexane	10.6		59.6		22.1		[39]
		ethyl acetate	11.2		Not evaluated		Not evaluated		
		ethanol	11.3		Not evaluated		Not evaluated		
14	Cold pressing and DCS	ethanol	Not evaluated		Not evaluated		Not evaluated		[54]
15	Ultrasound extraction	acetone	Not evaluated		Not evaluated		Not evaluated		[34]
16	Ultrasound liquid-liquid extraction	methanol-water solution	Not evaluated		Not evaluated		Not evaluated		[55]
17	Supercritical fluid extraction	CO_2_	88.1		63.4		35.8		[47]
18	Supercritical fluid extraction	CO_2_	7.2		66.0		18.2		[35]
19	Supercritical fluid extraction	CO_2_	10.6		62.3		19.7		[39]
20	Supercritical fluid extraction	CO_2_	27.8–31.8		52.5–55.9		19.8–20.9		[46]
21	Supercritical fluid extraction	CO_2_	17.5		Not evaluated		Not evaluated		[52]
22	Supercritical fluid extraction	ethanol	64.5–90.3		65.0–68.0		17.0–23.0		[21]
23	Supercritical fluid extraction (with/without ultrasound and cosolvent)	CO_2_	24.6		68.3		18.6		[56]
24	Subcritical fluid extraction	*n*-propane	Not evaluated		47.3	46.2	17.8	17.5	[9]
25	Pressing	*/*	20.3–24.8		64.5–69.3		16.6–17.5		[57]
26	Pressing	*/*	20.1		67.9		19.1		[58]
27	Pressurized liquid extraction	ethanol	17.7–19.9		65.0–68.0		17.0–23.0		[21]
28	Pressurized liquid extraction	*n*-hexane	Not evaluated		65.5		18.1		[59]
29	Screw pressing	*n*-hexane	9.5	9	Not evaluated	3.5	4.0	2.97	[23]
30	Seed compression	/	Not evaluated		Not evaluated		Not evaluated		[9]
31	Cold press and ultrasound	Methanol	Not evaluated		66.8–68.7		19.2–21.7		[22]
32	High pressure extraction	/	20.01		Not evaluated		Not evaluated		[52]
33	Alkaline extraction and isoelectric precipitation	/	Not evaluated		Not evaluated		Not evaluated		[32]
34	Ultrasound-assisted extraction	*n*-hexane	Not evaluated		Not evaluated		Not evaluated		[16]
36	Hot solvent extraction	Water and aqueous ethanol	Not evaluated		Not evaluated		Not evaluated		[60]

^a^ Highest amounts are presented.

**Table 4:** Clinical studies of the therapeutic value of the chia seeds.

Aim of the Study	Clinical Setting	Study Description	Result	Reference
Assessment of the effect of Salba-chia on body weight, visceral obesity and obesity-related risk factors in overweight and obese adults with type 2 diabetes.	- Changes in body weight and in waist circumference, - body composition, - glycemic control, - level of C-reactive protein and obesity-related satiety hormones.	- Two parallel groups with 77 over-weight or obese patients with type 2 diabetes were evaluated.	- Significant weight loss, - reduction in waist circumference and C-reactive protein - increase of plasma adiponectin.	[66]
Comparison of the effect of two seeds (flax (*Linum usitatissimum*) and Salba-chia (*Salvia hispanica L.*)) on postprandial glycemia and satiety scores.	Blood glucose samples and satiety ratings were collected at fasting and over 2 h postprandially.	- Fifteen healthy participants - randomized to receive a 50 g glucose challenge, alone or supplemented with either 25 g ground Salba-chia or 31.5 g flax.	- Salba-chia appears to have the ability to convert glucose into a slow-release carbohydrate - and affect satiety to a greater extent than flax (due to the higher fiber viscosity).	[67]
Influence of Ingesting Chia Seed Oil on Human Running Performance	- A randomized (1:1 allocation, random number generator), - crossover approach, and - subjects engaged in two run-to-exhaustion trials after acute ingestion of flavored water with chia seed oil or flavored water alone (no blinding), with at least a two-week washout period.	- After providing a blood sample at 8:00 am, subjects ingested 0.5 L flavored water alone or 0.5 L water with 7 kcal kg−1 chia seed oil (random order), provided another blood sample at 8:30 am, and then started running to exhaustion. - Additional blood samples were collected immediately post- and 1 h post-exercise.	- Ingestion ofchia seed oil 30 min before running caused an increase in plasma ALA levels, - no discernable benefits for the athletes in this study.	[75]
Effect of chia supplementation (Salvia hispanica L.) on blood pressure (BP) and its associated cardiometabolic factors.	- Hypertensive individuals of both sexes, –randomized, double-blind, experimental and placebo-controlled study.	- Nutritional assessment, -clinical BP measurement, - ambulatory blood pressure monitoring (ABPM), - collection of blood samples.	- The consumption of the chia or the placebo caused no gastrointestinal, hepatic or renal disorders, - decrease of the BP in hypertensive individuals.	[68]
Effectiveness of milled and whole chia seed in altering disease risk factors in overweight, postmenopausal women.	- Metabolomics approach using gas chromatography–mass spectrometry with multivariate statistical methods, - including principal component analysis and partial least-square discriminant analysis (PLS-DA).	- Subjects ingested 25 g chia seed or placebo supplements each day for 10 weeks, - body mass and composition, blood pressure and augmentation index, serum lipid profile, inflammation markers from fasting blood samples, plasma fatty acids, and metabolic profiling.	Ingestion of 25 g/day milled chia seed compared to whole chia seed or placebo for 10 weeks by overweight women increased plasma ALA and EPA, but had no influence on inflammation or disease risk factors using both traditional and metabolomics-based measures.	[76]
Evaluation of the effects of a dietary pattern (DP; soy protein, nopal, chia seed, and oat) on the biochemical variables of MetS, the AUC for glucose and insulin, glucose intolerance (GI), the relationship of the presence of certain polymorphisms related to MetS, and the response to the DP.	A single-center, randomized, placebo-controlled, double-blind, parallel-arm study.	- In the first stage, participants were instructed to consume a reduced energy diet according to (23) and a low-saturated fat and low-cholesterol diet for 2 wk (5). - During the second stage of the study, participants were randomly assigned to consume either the dietary pattern (DP) or placebo (P) in addition to the reduced energy diet for 2 mo.	- BW, BMI, and WC decreased, - no changes in the percentages of the lean or fat mass in either group after the dietary treatment.	[77]
Assessment of Omega 3 chia seed loading as a means of Carbohydrate loading.	-CHO-loading treatments were based on the subject’s body weight and were thus isocaloric.	Comparison of the performance testing results between 2 different CHO-loading treatments	- No statistical difference between Omega 3 Chia loading and CHO loading.	[78]

## Abbreviations

ABST^+^	Monocationic radical from ABTS diammonium salt
CO_2_	Carbon dioxide
DPPH	2,2-dyphenyl-1-picrylhydrazyl
FRAP	Fluorescence recovery after photobleaching
GC	Gas Chromatography
HPLC	High Performance Liquid Chromatography
DSC	Differential Scanning Calorimetry
SFC	Supercritical fluids
PLE	Pressured-liquid Extraction
UHLPC	Ultra-high Performance Liquid Chromatography
UPLC	Ultra-performance Liquid Chromatography
